# Extraembryonic but not embryonic SUMO-specific protease 2 is required for heart development

**DOI:** 10.1038/srep20999

**Published:** 2016-02-17

**Authors:** Eri O. Maruyama, Heng Lin, Shang-Yi Chiu, H.-M. Ivy Yu, George A. Porter, Wei Hsu

**Affiliations:** 1 Department of Biomedical Genetics, Center for Oral Biology, University of Rochester Medical Center, 601 Elmwood Avenue, Box 611, Rochester, NY 14642; 2 Stem Cell and Regenerative Medicine Institute, University of Rochester Medical Center, 601 Elmwood Avenue, Box 611, Rochester, NY 14642; 3 Wilmot Cancer Institute, University of Rochester Medical Center, 601 Elmwood Avenue, Box 611, Rochester, NY 14642; 4 Departments of Pediatrics, Pharmacology and Physiology, and Medicine, University of Rochester Medical Center, 601 Elmwood Avenue, Box 631, Rochester, NY 14642.

## Abstract

SUMO-specific protease 2 (SENP2) activities to remove SUMO from its substrates is essential for development of trophoblast stem cells, niches and lineages. Global deletion of SENP2 leads to midgestation lethality, and causes severe defects in the placenta which is accompanied by embryonic brain and heart abnormalities. Because of the placental deficiencies, the role of SENP2 in development of the embryonic tissues has not been properly determined. The brain and heart abnormalities may be secondary to placental insufficiency. Here we have created a new mouse strain permitting conditional inactivation of *SENP2*. Mice homozygous for germline deletion of the conditional allele exhibit trophoblast defects and embryonic abnormalities resembling the global SENP2 knockout. However, tissue-specific disruptions of SENP2 demonstrate its dispensable role in embryogenesis. Placental expression of SENP2 is necessary and sufficient for embryonic heart and brain development. Using a protease deficient model, we further demonstrate the requirement of SENP2-dependent SUMO modification in development of all major trophoblast lineages. SENP2 regulates sumoylation of Mdm2 which controls p53 activities critical for G-S transition of mitotic division and endoreduplication in trophoblast proliferation and differentiation, respectively. The differentiation of trophoblasts is also dependent on SENP2-mediated activation of p57^Kip2^, a CDK-specific inhibitor required for endoreduplication.

Small ubiquitin-related modifier (SUMO), regulating posttranslational modification of proteins, is a member of the ubiquitin-like modifier family^1^. SUMO modification is reversible and highly evolutionary conserved from yeasts to humans^2^. SUMO modification is involved in a variety of cellular processes, including protein trafficking, cell cycle, cell survival and death^3^,^4^,^5^,^6^,^7^. The conjugation of proteins by SUMO has been shown to alter their function, activity, interaction with other proteins, and subcellular distribution. Similar to ubiquitination, sumoylation requires processing, conjugation and transfer. The transfer process, which covalently conjugates SUMO polypeptides to their targets, is catalyzed by E3 ligases^1^,^8^. The reverse desumoylation process is mediated by SUMO proteases^9^,^10^. The hallmark of these proteases is the highly conserved SENP domain of ~200 amino acids located at the carboxyl terminus. Only certain SENPs possess the hydroxylase activity required for SUMO peptide maturation, but all SENPs are isopeptidases. They catalyze desumoylation in various physiological systems, and genetic analysis has recently begun to unfold their importance in mammalian development and disease^11^,^12^,^13^,^14^.

Genetic inactivation of *SENP2* in mice reveals its essential role in development of trophoblast stem cell niches and lineages during placental formation^11^. Although mice with global deletion of SENP2 also exhibit defects in the embryonic brain and heart^12^,^13^, the placental insufficiency greatly complicates analysis of these phenotypes^11^. As these defects arise at specific stages of the embryonic development when organogenesis becomes highly dependent on placental function, it is possible that the brain and heart abnormalities of the SENP2 global knockout are not primary defects, but are secondary to placental insufficiency. Thus, the essential role of SENP2 in brain and heart development requires further investigation. This hypothesis has recently been proved for analysis of embryonic brain development^13^. Conditional ablation of SENP2 in the neural progenitor cells does not result in brain abnormalities shown in the global knockouts^13^. With a healthy placenta containing intact SENP2, embryos with neural-specific deletion of SENP2 display fairly normal brain, demonstrating the contribution of placental insufficiency to the observed embryonic deformities. Although required for postnatal brain development, SENP2 is dispensable in the neural cells during embryogenesis. However, it remains unknown if the cardiac defects observed in the SENP2 global knockout embryos are also secondary to placental deficiencies.

## Results

### Creation of a conditional null allele for SENP2

**Figure 1:**
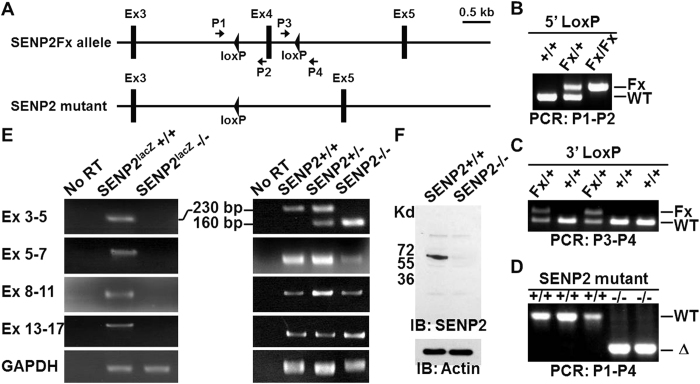
Diagrams illustrate the targeting strategy and the creation of mice carrying SENP2Fx or SENP2 mutant allele. (**A**) In the targeted allele, a loxP site and a pgk-neo cassette flanked by two loxP sites were inserted into intron 3 and intron 4, respectively. Mice carrying the SENP2 targeted allele were created, and crossed with the EIIa-Cre transgenic mice to generate progeny carrying the SENP2Fx or SENP2 mutant allele. (**B–D**) PCR analysis detected the presence of 5′ (PCR: P1–P2) and 3′ (PCR: P3–P4) loxP sites for genotyping the wild type (+/+) and heterozygous (Fx/+) mice, and examined the deletion of exon 4 (PCR: P1–P4). (**E**) RT-PCR analysis detected the transcripts generated from the wild type (+/+), heterozygous (+/−) and homozygous (−/−) embryos of SENP2^lacZ^ (left panel) and SENP2 mutant (right panel). (**F**) Immunoblot analysis examines protein expression in SENP2+/+ and SENP2−/− embryos. Actin level is used as a loading control.

To determine the requirement of SENP2 in embryonic development, we decided to generate a conditional null allele of *SENP2* in mice. This new SENP2Fx mouse strain, permitting Cre-mediated deletion of SENP2, was created by insertion of two loxP sites flanking exon 4 (Fig. 1A). The integration of 5′ and 3′ loxP sites into the mouse genome was further identified by PCR analysis (Fig. 1B,C). Using EIIa-Cre to mediate site-specific recombination in the germ cells, we also obtained a mouse strain carrying deletion of the exon 4 (Fig. 1D). RT-PCR analysis then revealed the expected SENP2 RNA transcribed from wild type, SENP2^lacZ^ knock-in^11^ and the newly created SENP2 mutant alleles (Fig. 1E). Due to insertion of a lacZ reporter into the second coding exon, no signal was detected in the SENP2^lacZ^−/− embryo. In the SENP2−/− embryos, we were able to identify correct transcript containing all exons except for exon 3–5. The deletion of exon 4 not only reduced the size of this RT-PCR product but also created an out of frame deletion (Fig. 1E). Although the mRNA was transcribed in these mutants, no protein could be detected (Fig. 1F), thus confirming SENP2Fx as a conditional null allele.

### The SENP2 nulls exhibit similar placental and heart defects shown in SENP2^lacZ^ mutants

**Figure 2:**
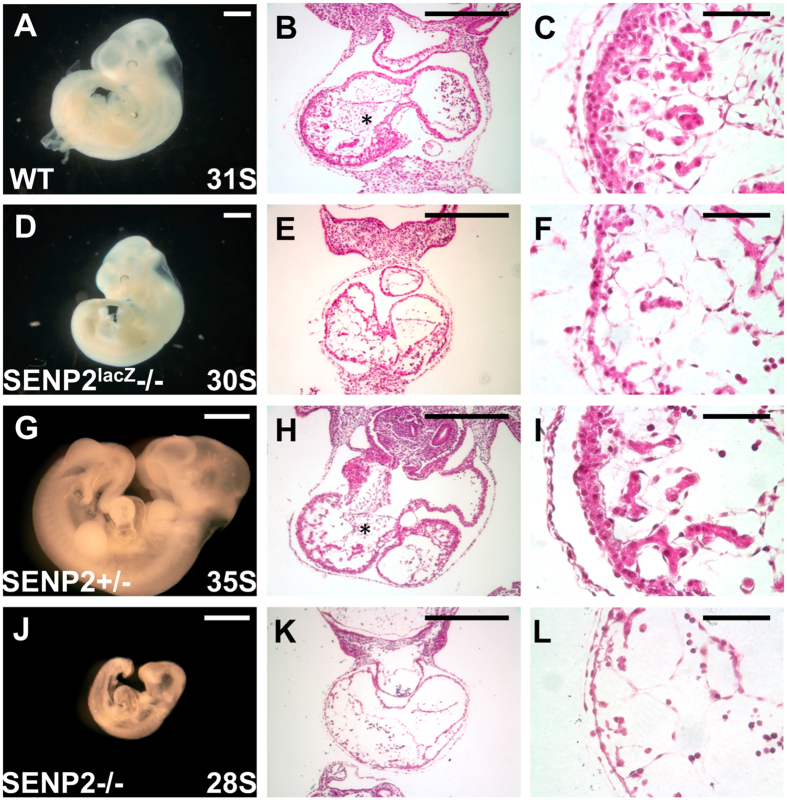
Heart development is deformed in the SENP2 homozygous embryos similar to those of SENP2^lacZ^. Gross morphological evaluation of the wild type (**A**), SENP2^lacZ^−/− (**D**), SENP2+/− (**G**) and SENP2−/− (**J**) embryos at the specific somite stage (S) as indicated identifies growth restriction caused by the deletion of SENP2. Histology shows the atrioventricular (AV) cushion (**B,E,H,K;** asterisks) and myocardium (**C,F,I,L**) defective in the mutants (**E,F,K,L**). Scale bars, 1 mm (**A,D,G,J**); 500 μm (**B,E,H,K**); 100 μm (**C,F,I,L**).

**Figure 3:**
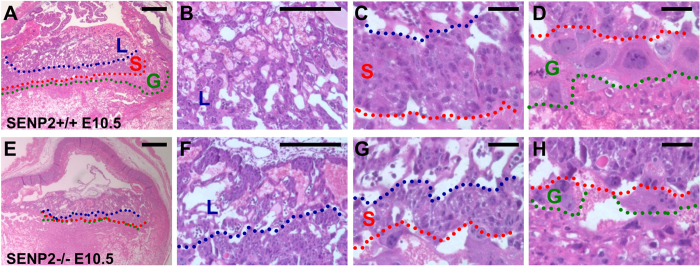
The SENP2 homozygous mutant exhibits extraembryonic abnormalities. Histology examines the placentas of SENP2+/+ (**A–D**) and SENP2−/− (**E–H**) in transverse sections at E10.5. Labyrinth (L), spongiotrophoblast (S) and trophoblast giant cell (G) layers were defined by blue, red and green broken lines, respectively. Scale bars, 500 μm (**A,E**); 50 μm (**B–D,F–H**).

We next examined the phenotypes associated with removal of exon 4 for *SENP2*. Intercross of SENP2 +/− mice resulted in the homozygous embryos which appear underdeveloped at E9.5–10.5 (Fig. 2A,D,G,J) and died ~E11.5. The SENP2−/− placentas were smaller and paler than the controls. Histological evaluations revealed severe abnormalities in all three major trophoblast layers (Fig. 3). The trophoblast giant cell (TGC) layer most severely affected by the mutation is almost completely missing. These abnormalities phenotypically copy those detected in the SENP2^lacZ^ homozygote, suggesting that they faithfully reflect the effects caused by global disruption of SENP2. Furthermore, development of the embryonic heart was affected in these two different SENP2 mutant strains (Fig. 2) similar to a previous report^12^. The mutant cardiac chambers were generally smaller with pericardial effusion. Histological sections showed marked myocardial thinning (Fig. 2C,F,I,L) and missing of atrioventricular (AV) cushions (Fig. 2B,E,H,K).

### Endothelial deletion of SENP2 does not cause endocardial cushion defects

The endocardium is vital to both AV cushion and myocardial development. In particular, AV cushion mesenchyme is derived from local endocardium via epithelial-mesenchymal transition (EMT). The process is controlled by a regulatory loop of signaling factors secreted by both tissues which activate downstream effectors^15^. The severe defects in the development of both the AV cushion and the myocardium caused by SENP2 deficiency prompted us to examine its role in the endocardium. We therefore deleted SENP2 in the endothelial cells using Tie2-Cre which efficiently promotes site-specific recombination in the endocardium and the endothelial and mesenchymal cells in the AV cushion (Supplementary Figure S1A–H). Mice carrying Tie2-Cre transgene or SENP2Fx allele were intercrossed to generate the Tie2-Cre; SENP2Fx/Fx mutants (SENP2^Tie2^). Histological evaluations revealed no obvious difference between the control and SENP2^Tie2^ heart at E10.5 (Supplementary Figure S1I–P). We were able to obtain the SENP2^Tie2^ newborns which are alive (Supplementary Table S1), suggesting that SENP2 is dispensable in the endothelial lineage during embryonic heart formation.

**Figure 4:**
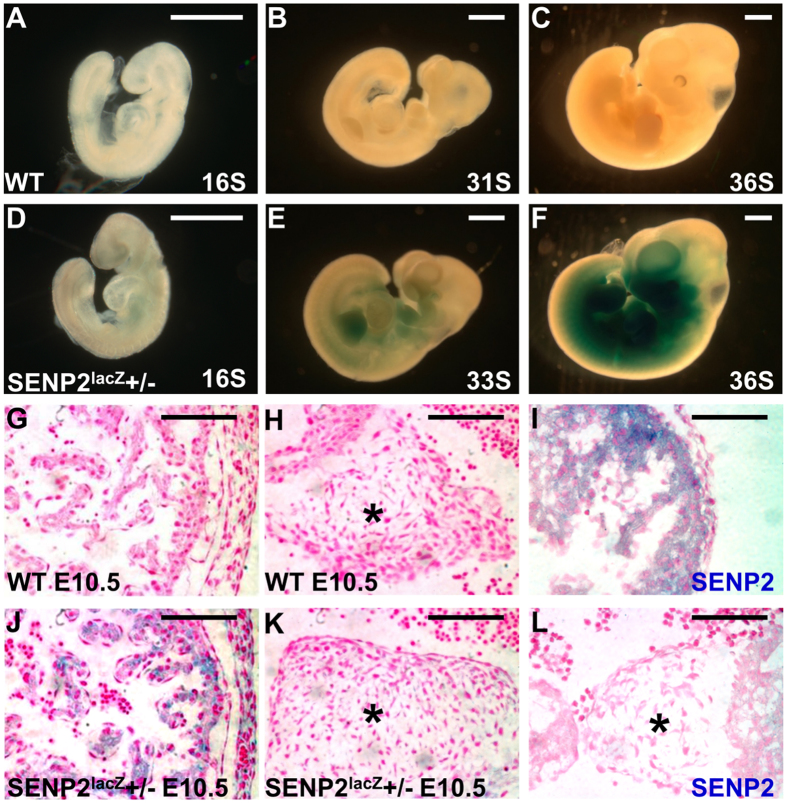
SENP2 is expressed in developing embryonic heart. The expression pattern of SENP2 is examined in control (**A–C,G–I**) and SENP2^lacZ^ heterozygous (**D–F,J–L**) embryos by β-gal staining and *in situ* hybridization (**I,L**) in whole mounts (**A–F**) and sections (**G–L**). Asterisks indicate AV cushions. Scale bars, 1 mm (**A–F**); 100 μm (**G–L**).

We examined whether this is attributed to its lack of expression in the endocardial cells using the SENP2^lacZ^ allele and *in situ* hybridization. Whole mount β-gal staining showed no detectable signal at E9 (Fig. 4A,D). At E10, SENP2 began to be expressed in nasal, mandibular and maxillary processes, as well as fore limb and hind limb buds (Fig. 4B,E). At E10.5, the expression pattern was expanded to other regions, including the heart (Fig. 4C,F), specifically in the myocardium (Fig. 4G,J). *In situ* hybridization analysis showed that SENP2 is mainly expressed in the myocardium with very low signals detected in the AV cushion (Fig. 4I,L). Therefore, the myocardial and AV cushion defects observed in the SENP2^lacZ^−/− and SENP2−/− (Fig. 2) are not due to the expression of SENP2 in the endocardium, which is known to regulate the formation of both tissues. It appears to be a secondary effect, possibly due to deficiencies of the myocardium or in other embryonic or extraembryonic tissues.

### Heart defects in the global knockout of SENP2 are caused by placental insufficiency

**Figure 5:**
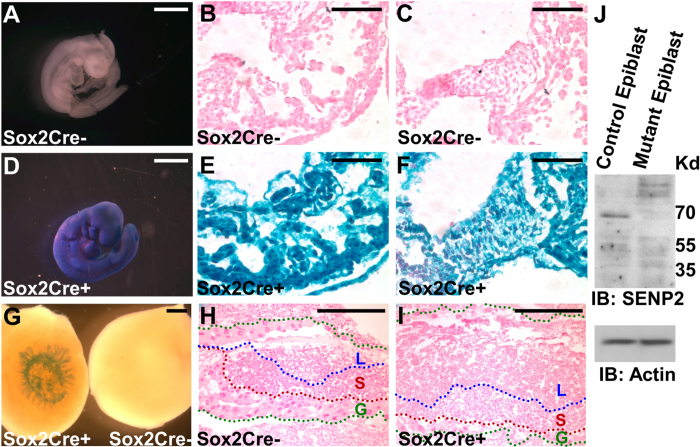
Sox2-Cre permits loxP site-specific recombination in the embryonic but not extraembryonic tissues. The efficiency of the Sox2-Cre mediated recombination in the epiblast, including myocardium (**B,E**) and AV cushion (**C,F**), and trophoblast (**H,I**) is analyzed by β-gal staining in whole mounts (**A,D,G**) and sections (**B,C,E,F,H,I**) of the R26RlacZ heterozygous embryo (**A–F**) and placenta (**G–I**), negative (**A–C,G–H**) or positive (**D–F,G,I**) for the Cre transgene. Note positive β-gal stains in the left placenta in panel G are attributed to presence of the residual embryonic tissue/yolk sac. Sections were counterstained by nuclear fast red. Labyrinth (L), spongiotrophoblast (S) and trophoblast giant cell (G) layers were defined by blue, red and green broken lines, respectively. (J) Immunoblot analysis examines protein expression in the control and SENP2 mutant epiblasts. Actin level is used as a loading control. Scale bars, 1 mm (**A,D**); 500 μm (**B,C**,**E–I**); 800 μm (G).

**Figure 6:**
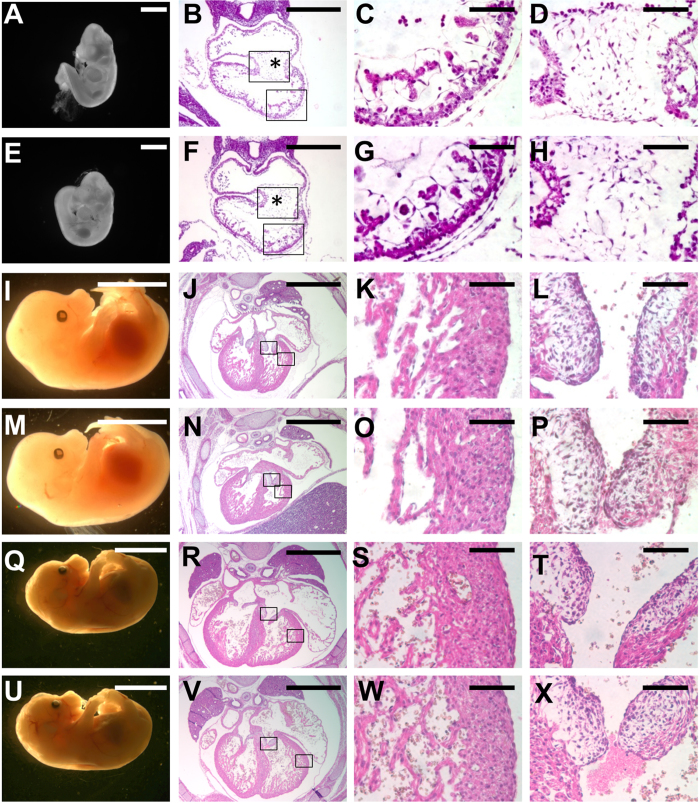
SENP2 is dispensable for embryogenesis. The E10.5 (**A–H**), E14.5 (**I–P**) and E18.5 (**Q–X**) control (**A–D,I–L,Q–T**, genotype: SENP2Fx/Fx) and SENP2^Sox2^ mutant (**E–H,M–P**, genotype: Sox2-Cre+; SENP2Fx/Fx and **U-X**, genotype: Sox2-Cre+; SENP2Fx/−) embryos were examined in whole mounts and H&E stained sections. Asterisks indicate AV cushions and enlargements of the inset are shown in (**C,D,G,H,K,L,O,P,S,T,W,X**). Scale bars, 1 mm (**A,E**); 5 mm (**I,M,Q,U**); 500 μm (**B,F,J,N,R,V**); 100 μm (**C,D,G,H,K,L,O,P,S,T,W,X**).

To determine if the cardiac defects observed in the SENP2 knockouts were due to myocardial deletion or placental deletion of this gene, we deleted SENP2 in the embryo but not the placenta using Sox2-Cre line, which promotes recombination in epiblast cells (Fig. 5). By crossing Sox2-Cre with an R26RlacZ reporter line, we found highly efficient recombination which occurs only in the embryonic tissues, including the myocardium and AV cushion (Fig. 5A–F). No Cre activity could be detected in any of the three trophoblast layers during placentation (Fig. 5G–I), demonstrating Sox2-Cre is able to mediate gene deletion specifically for the embryonic but not extraembryonic tissues. Next, we generated mutants carrying Sox2-Cre; SENP2Fx/Fx (SENP2^Sox2^) in which SENP2 is specifically deleted in the embryonic but not extraembryonic tissues (Fig. 6). Immunoblot analysis showed effectiveness of the SENP2 ablation in the mutant epiblasts (Fig. 5J). Surprisingly, we were able to identify the SENP2^Sox2^ mutants at E10.5 and E14.5 without any noticeable gross abnormality (Fig. 6A,E,I,M). The ventricular myocardium (Fig. 6C,G,K,O), AV cushion (Fig. 6D,H), tricuspid valve and mitral valve (Fig. 6L,P) of SENP2^Sox2^ also appear to be normal (Fig. 6B–D,F–H,J–L,N–P). Furthermore, SENP2^Sox2^ mice were viable at birth. To ensure that the lack of embryonic defects in this genetic study is not attributed to inefficient recombination required for excising two floxed alleles, we generated mutants carrying Sox2-Cre; SENP2Fx/- in which one allele of SENP2 has already been deleted. No structural defects were also found in these mutants at E18.5 (Fig. 6Q–X), suggesting that SENP2 is dispensable for not only heart development but also embryogenesis. Therefore, we concluded that the heart deformities of SENP2 global knockouts are secondary due to placental insufficiency.

### SUMO modification is essential for development of three major trophoblast lineages

**Figure 7:**
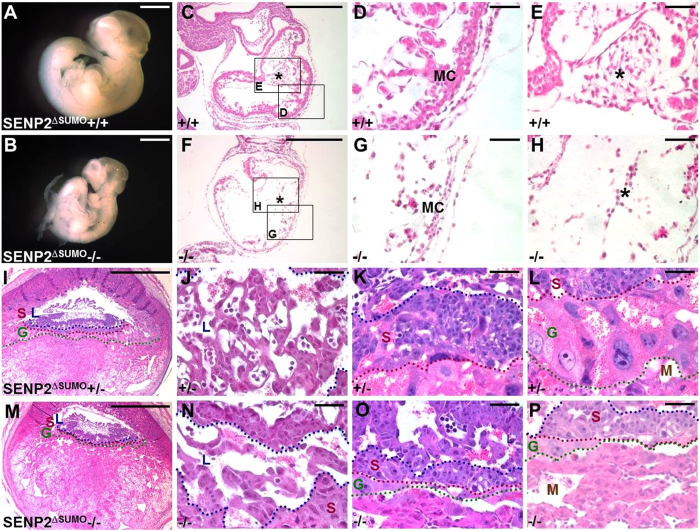
The SUMO protease core domain of SENP2 is essential for normal placentation leading to development of a healthy embryo. The E10.5 control (1^st^ and 3^rd^ rows, genotype: SENP2^∆SUMO^+/+ or +/∆) and protease core domain-deficient mutant (2^nd^ and 4^th^ rows, genotype: SENP2^∆SUMO^−/−) embryos (**A–H**) and placentas (**I–P**) were examined in whole mounts (**A,B**) and H&E stained sections (**C–P**). Enlargements of the insets (**C,F**) are shown in D, E, G and H. Labyrinth (L), spongiotrophoblast (S) and trophoblast giant cell (G) layers were defined by blue, red and green broken lines, respectively. M, MC and asterisk indicate maternal decidua, myocardium and AV cushion, respectively. Scale bars, 1 mm (**A,B,I,M**); 500 μm (**C,F**); 50 μm (**D,E,G,H,J–L,N–P**).

In this study, we showed that disruption of SENP2 causes placental deformities in which trophoblast stem cell niches and all three major lineages are defective using two different mouse strains (Fig. 3), which is in agreement with previous published data^11^. Although SUMO protease is the only biochemical activity so far found in SENP2, it is a large protein which may possess additional function. To examine if the placental defects are associated with SUMO protease activity, we used another mouse allele where only the protease core domain is deleted^13^. Because of an in-frame deletion, the SENP2^∆SUMO^ allele produced a truncated, protease deficient protein^13^, thus permitting a rigorous genetic analysis. We detected congenital deformities in the SENP2^∆SUMO^ homozygous hearts and placentas (Fig. 7), similar to the SENP2^lacZ^−/− and SENP2−/− mutants^11^ (Fig. 2). These results strongly suggest the observed deformities are associated with SUMO modification deficiency.

### The SENP2-Mdm2-p57^Kip2^ regulatory axis in TGC development

**Figure 8:**
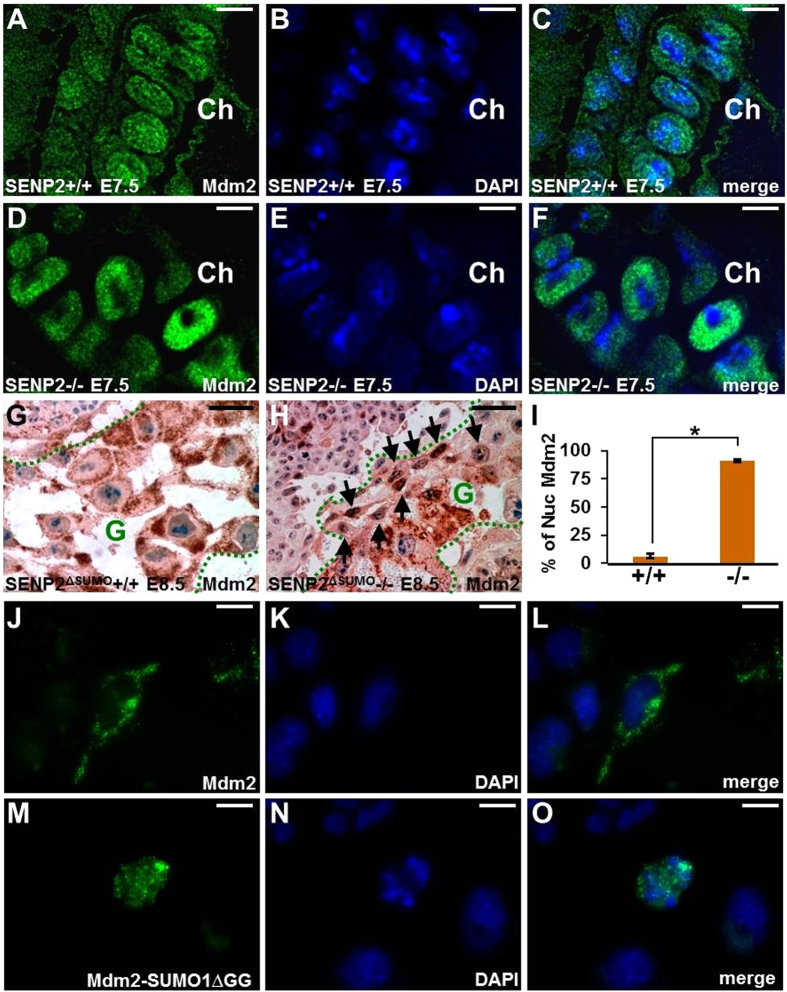
SENP2-mediated SUMO modification regulates subcellular distribution of Mdm2. Immunostaining of Mdm2 was performed on the SENP2+/+ (**A–C,G**) and SENP2−/− (**D–F,H**) placentas at E7.5 (**A–F**) and E8.5 (**G,H**). Sections were counterstained with DAPI (**B–C,E–F**) or hematoxylin (**G,H**). (**I**) Statistical analysis indicates the percentage of nuclear (Nuc) vs. non-nuclear localizations of Mdm2 (data represent the mean ± SEM; *p < 0.000001, n = 3). Three samples were used and 3–4 sections from each sample were counted. GFP analysis of TS cells, transfected by the GFP tagged Mdm2 (**J–L**) or Mdm2-SUMO1∆GG (**M–O**), reveals their differential compartmentalization. Arrows indicate dislocation of Mdm2 from the cytoplasm to nucleus of TGC. Ch, chorion; G, TGC layer. Scale bars, 20 μm (**A–F,J–O**); 50 μm (**G,H**).

SENP2 was shown to regulate the G-S transition, which is required for mitotic and endoreduplication cell cycles in trophoblast proliferation and differentiation, respectively, through modulation of the p53-Mdm2 pathway^11^. Next, we examined if subcellular distribution of Mdm2 was also affected in the SENP2−/− and SENP2^∆SUMO^−/− mutants similar to the previous observation using the SENP2^lacZ^ allele^11^. In the trophoblast stem cell niche, Mdm2 exhibited both nuclear and cytoplasmic staining (Fig. 8A–C). However, nuclear accumulation was predominantly shown in the mutant (Fig. 8D–F). In the differentiated TGC cells, Mdm2 mainly showed cytoplasmic localization (Fig. 8G,I; nucleus vs. non nucleus = 6.07% vs. 93.93% ± 2.48, p < 0.000001, n = 3, a total of ~570 cells counted). The removal of the SUMO protease core domain alters Mdm2 accumulation predominantly to the nucleus (Fig. 8H,I; nucleus vs. non nucleus = 91.24% vs. 8.76% ± 0.80, p < 0.000001, n = 3, a total of ~540 cells counted), suggesting that its subcellular distribution is modulated by sumoylation. We previously showed that SENP2 regulates sumoylation of Mdm2 to modulate p53 activities essential for trophoblast proliferation and differentiation^11^. To further examine whether SUMO modification alters cellular compartmentalization of Mdm2, TS cells were transiently expressed GFP tagged Mdm2 or Mdm2-SUMO1∆GG. The ∆GG mutation removes two glycine residues in SUMO1 essential for substrate modification. Their presence may interfere with our analyses due to conjugation of the Mdm2-SUMO1 chimeric protein to other molecules which may have intrinsic signal sequences and preferences in cellular compartmentalization. This removal prevents further conjugation thus permitting an assessment on Mdm2 localization affected by SUMO1 conjugation. The results clearly demonstrated that SUMO1 conjugation of Mdm2 promotes its nuclear localization (Fig. 8J–O), demonstrating the importance of the SENP2-mediated SUMO modification in the regulation of Mdm2.

**Figure 9:**
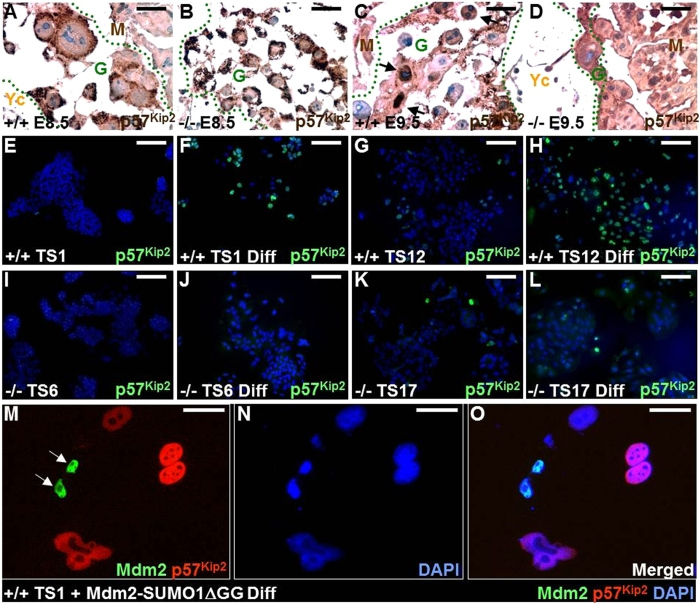
SENP2 regulation of cyclin-dependent kinase inhibitor 1C through SUMO modification of Mdm2 is essential for trophoblast stem cell development. Immunostaining of Cyclin-dependent kinase inhibitor 1C (p57^Kip2^) was performed on E8.5 (**A,B**) and E9.5 (**C,D**) SENP2+/+ (**A,C**) and SENP2−/− (**B,D**) placentas. Positive staining of p57^Kip2^ identifies differentiated TGCs in four independent trophoblast stem cell lines (Wild type: TS1, TS12; Mutant: TS6, TS17) before (**E,G,I,K**) and after (**F,H,J,L,M–O**) induction of differentiation. (**M–O**) Wild type TS1 trophoblast stem cells transfected with a construct expressing GFP-tagged Mdm2-SUMO1∆GG were differentiated into TGCs, followed by immunostaining of p57^Kip2^, DAPI counterstaining and imaging analysis. G, TGC layer (primary TGC in **A–B** and secondary TGC in **C–D**); M, maternal decidua; Yc, yolk sac. Scale bars, 50 μm (**A–D,M–O**); 200 μm (**E–L**).

The differentiation of TS cells into polyploid TGCs is mediated by endoreduplication, which is triggered by p57^Kip2^ mediated inhibition of CDK1 to regulate the G-S transition^16^. We therefore examined if p57^Kip2^ acts downstream of the SENP2-Mdm2 signaling pathway. *In vivo*, p57^Kip2^ localized predominantly to the cytoplasm at E8.5, but upon further differentiation (e.g. E9.5), it was localized to the nucleus (Fig. 9A,C). However, this distribution switch did not effectively occur in the SENP2−/− TGCs (Fig. 9B,D). Using TS cell lines derived from the wild type and knockout blastocysts, we further examined the regulation of p57^Kip2^ by SENP2. In the cultured TS cells, nuclear accumulation of p57^Kip2^ became clearly evident upon differentiation (Fig. 9E–H). The loss of SENP2 greatly abrogated this regulatory event (Fig. 9I–L), indicating the importance of SUMO modification in TGC differentiation. To further assess if the regulation of p57^Kip2^ by SENP2 is mediated through SUMO modulation of Mdm2, we examined the effects of sumoylated Mdm2 on TS cell differentiation. The wild type TS cells were transfected to express Mdm2-SUMO1∆GG, followed by immunostaining of p57^Kip2^ (Fig. 9M–O). Cells expressing Mdm2-SUMO1∆GG were excluded from p57^Kip2^ expression (Fig. 9M), suggesting that SUMO conjugation of Mdm2 prohibits the p57^Kip2^ mediated cell cycle regulation/endoreduplication. The SENP2-Mdm2- p57^Kip2^ regulatory axis is essential for trophoblast development.

## Discussion

Genetic analyses described in this study demonstrate a dispensable role of SENP2 in embryonic heart development. SENP2 is also not required for prenatal brain development and embryogenesis in general. Kang *et al*. has previously reported embryonic heart malformations, including development of the myocardium and AV cushion, caused by insertion of a gene trapped vector into intron 10 of *SENP2*, suggesting its essential function during heart development^12^. Although this report does not describe any trophoblast abnormality, impaired placentation is highly suspected in the gene trap mutants as well. This speculation remains to be addressed but is supported by various types of SENP2 mutation in mice. Three different mutant alleles, SENP2^lacZ^, SENP2^null^ and SENP2^∆SUMO^, which we have generated by targeted mutagenesis, also cause very similar heart defects when the deletion occurs globally. However, deletion of SENP2 in the epiblasts does not cause noticeable embryonic defects and these mutants are viable at birth, indicating SENP2 is not essential for embryogenesis.

Our findings here strongly argue against a specific requirement of SENP2 in cardiac tissue during heart development as we find that the heart deformities associated with global inactivation of *SENP2* are not primary but secondary defects due to placental insufficiency. Whether the described pathogenic mechanism associated with polycomb group proteins mediated gene silencing^12^ plays a role in cardiovasculogenesis at all remains unclear. However, this regulatory pathway is most likely not required for heart development. The early implanted embryo relies on diffusion of nutrients and wastes from the maternal tissues but its survival requires intact placental and embryonic circulation after about E10^17^. During the development of normal circulation, myocardial development and cardiac morphogenesis depend on the patterns of blood flow streams returning from the extraembryonic tissues^18^. Therefore, placental abnormalities in the SENP2 mutants likely disrupt cardiac development by altering hemodynamic forces of blood returning to the heart, perhaps through modulation of pathways responsive to shear stress^18^. Finally, it is possible that SENP2-mediated SUMO modification is crucial for healthy development of the heart at postnatal stages, but this will require further investigation.

SENP2 regulates SUMO modification of Mdm2 which controls p53 activities critical for G-S transition of the cell cycle^11^. Both mitotic division and endoreduplication are affected by dysregulation of this regulatory axis. In contrast, the CDK inhibitor p57^Kip2^ is only required for endoreduplication during trophoblast differentiation. The results imply that this inhibitor controlling the G-S transition acts downstream of SENP2-Mdm2. Furthermore, this regulatory axis is mediated by a specific isoform of SENP2 necessary and sufficient to negatively modulate the p53-dependent transcription and stress responses^19^. As a key determinant of p53 in human cancer, SENP2 is likely to be involved in tumorigenesis. A closely related SUMO-specific protease, SENP1, has also been shown to affect tumor cell growth with G1 arrest through modulation of CDK inhibitors^20^. Sumoylation has also been suggested to promote a transcriptional switch from repression to activation for Myc, essential for tumorigenesis^21^. Inhibition of the SUMO pathway therefore has a potential for cancer therapy^22^. Further analysis is necessary to determine the oncogenic role of SENP2 whose function in cell cycle check point may be causative to malignant transformation.

## Methods

### Mouse strains

The SENP2Fx ES cell lines were generated by electroporation of a targeting vector, containing the insertion of a loxP site in intron 3 and a pgk-neo cassette flanked by two loxP sites in intron 4, into CSL3 ES cells^11^,^13^,^23^,^24^. Two independent clones heterozygous for the targeted allele were injected into blastocysts to generate chimeras which were bred to obtain mice carrying the targeted allele. These mice were then crossed with the EIIa-Cre transgenic mice to remove the pgk-neo cassette with or without the deletion of exon 4 to obtain the SENP2Fx or SENP2 null (-) mouse strain, respectively. Mice were genotyped by PCR analysis using primers (P1: 5′-CAAGAAACCTAACCACACCTATGTC-3′, P2: 5′-CATGATTATTTCAGCTAGCACACAG-3′) to identify the 5′ loxP locus, primers (P3: 5′-ACAGGAAGGATGTTTAACCCAGAGC-3′, P4: 5′-ATGCATATATGAGCCTGTGTGTGGA-3′) to identify the 3′ loxP locus and primers (5′-TCCAGCTTCTCCAAGAAACCTAACC-3′and 5′-CTCATGACCATTAGTGTGCAGTGCT-3′) to identify the exon 4 deleted locus. The SENP2^lacZ^, SENP2^ΔSUMO^Fx, SENP2^ΔSUMO^, Tie2-Cre and Sox2-Cre mouse strains and genotyping methods were reported previously^11^,^13^,^25^,^26^. Care and use of experimental animals described in this work were approved by and comply with guidelines and policies of the University of Committee on Animal Resources at the University of Rochester.

### Histology, β-gal staining

Samples were fixed, paraffin embedded, sectioned and stained with hematoxylin/eosin for histological evaluation^11^,^27^. Details for β-gal staining in whole mounts and sections were described previously^23^,^27^,^28^,^29^. Images were taken using Nikon SMZ1500 or TS100-F microscope (Nikon, Melville, NY, USA) equipped with a SPOT Pursuit Slider or Insight Camera (Diagnostic Instruments, Sterling heights, MI, USA).

### *In situ* hybridization, Immunostaining, Immunoblot analysis

*In situ* hybridization was performed as described^11^,^30^,^31^,^32^. In brief, sections were incubated with the digoxygenin labeled RNA probes generated by *in vitro* transcription^11^,^31^, followed by recognition with an alkaline phosphatase conjugated anti-digoxygenin antibody, and visualization with BM-purple^30^,^31^,^32^. Immunostaining of cells^11^,^33^ and tissue sections^11^,^34^,^35^,^36^ were performed by incubation with primary antibodies, followed by detection with fluorescence-conjugated or horseradish peroxidase-conjugated secondary antibodies. Images were taken using Zeiss Axio Observer microscope equipped with deconvolution analysis^19^,^31^,^36^. Immunoblot was performed by isolation of protein extracts from E9.5 embryos using M-PER (Pierce) in the presence of protease inhibitor cocktail, followed by electrophoresis as described^19^,^30^,^34^. Mouse monoclonal antibodies, Actin (Thermo Fisher), Mdm2 (Santa Cruz), p57^Kip2^ (Thermo Fischer); rabbit polyclonal antibody, SENP2 (Abgent) were used as primary antibodies as indicated.

## Additional Information

**How to cite this article**: Maruyama, E. O. *et al*. Extraembryonic but not embryonic SUMO-specific protease 2 is required for heart development. *Sci. Rep.* **6**, 20999; doi: 10.1038/srep20999 (2016).

## Supplementary Material

Supplementary Information
