# Supplementary material for: Extraembryonic but not embryonic SUMO-specific protease 2 is required for heart development
Source: Sci Rep. 2016 Feb 17;6:20999. doi: 10.1038/srep20999 (PMC4756675; doi:10.1038/srep20999)
Supplement: Supplementary Information [file srep20999-s1.pdf]

SUPPLEMENTARY INFORMATION

Extraembryonic but not embryonic SUMO-specific protease 2 is required for heart development

Eri O Maruyama[1], Heng Lin[1], Shang-Yi Chiu[1, 5], H-M Ivy Yu[1], George A Porter, Jr.[4], and Wei Hsu[1, 2, 3, 6]

[1]Department of Biomedical Genetics, Center for Oral Biology, [2]Stem Cell and Regenerative Medicine Institute, [3]Wilmot Cancer Institute, [4]Departments of Pediatrics, Pharmacology and Physiology, and Medicine, University of Rochester Medical Center, 601 Elmwood Avenue, Box 611, Rochester, NY 14642

[6]Corresponding Author
Phone 585-275-7802
Email Wei_Hsu@urmc.rochester.edu*

Figure S1. Heart development is not affected by endocardium-specific deletion of SENP2. The efficiency of Tie2-Cre to execute loxP-mediated recombination in the endocardium, including AV cushion, is analyzed by β-gal staining in whole mounts (A, E) and sections (B-D, F-H) of the R26RlacZ heterozygous embryo, negative (A-D) or positive (E-H) for the Cre transgene. The developmental stage of the embryos examined is indicated by the number of somite (S). Whole mount (I, M) and histological (J-L, N-P) evaluations examine heart development affected by Tie2-Cre mediated deletion of SENP2 (genotype for control: SENP2Fx/Fx, genotype for $SENP2^{Tie2}$ mutant: Tie2-Cre+; SENP2Fx/Fx). Asterisks indicate AV cushions and enlargements of the inset are shown in C, G, K, O (ventricular myocardium), and D, H, L, P (AV cushions). D, dorsal; V, ventral; R, right; L, left. Scale bars, 1 mm (A, E, I, M); 500 µm (B, F, J, N); 100 µm (C-D, G-H, K-L, O-P).

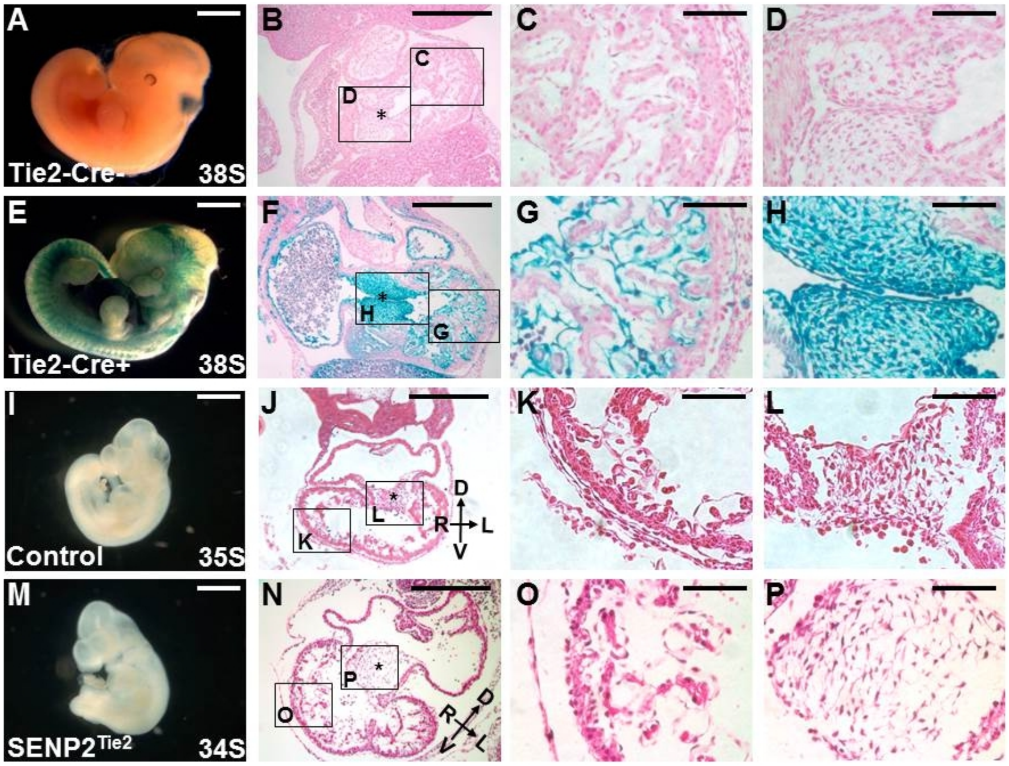

Table S1. Intercross of Tie2Cre+; Senp2Fx/+ and Senp2Fx/Fx generates live newborns with endocardium-specific deletion of SENP2.

Genotypes:	Tie2Cre	SENP2	No. Pups
1.	+	Fx/+	4
2.	+	Fx/Fx	5
3.	-	Fx/+	4
4.	-	Fx/Fx	3
